# Challenges and unmet needs of mothers of preschool children with autism spectrum disorders in Tunisia: a qualitative study

**DOI:** 10.11604/pamj.2022.43.66.36591

**Published:** 2022-10-07

**Authors:** Nihed Abid, Asma Ben Hassine, Naoufel Gaddour, Sihem Hmissa

**Affiliations:** 1 Department of Cytopathology, and Epidemiology of Cancer Research in the Tunisian Center, Faculty of Medicine of Sousse, University of Sousse, Sousse, Tunisia,; 2 Faculty of Nursing, Laval University, Quebec, Canada,; 3 Department of Child Psychiatry Service, Faculty of Medicine of Monastir, University of Monastir, Monastir, Tunisia

**Keywords:** Autism spectrum disorder, parenting, mother, perceived need, child with ASD, qualitative research, preschooler

## Abstract

**Introduction:**

Autism spectrum disorder (ASD) is a life-changing condition, not only for the child but also for the mother and the usual caregiver. In fact, a child recently diagnosed with ASD is a real challenge to mothers´ adaptation, involves their resources, and gives rise to a set of needs. This study explores the unmet needs and experiences of mothers of ASD children in the Tunisian context.

**Methods:**

a qualitative phenomenological design was chosen for this study and a semi-structured interview was used for eight mothers raising an autistic preschooler child.

**Results:**

the results indicate significant denial and rejection following the announcement of the diagnosis. To cope with this, reliance on religion has helped foster acceptance. Although informal support (from family and friends) has sometimes been mentioned, an increased need for training, social and financial support has been expressed and is a major concern given the high cost of TSA services.

**Conclusion:**

this study provides a deeper understanding of mothers' needs following the announcement of the diagnosis of ASD. These unmet needs should be taken into account when designing interventions strategies for children with ASD to help mothers cope and parent a child with ASD.

## Introduction

Autism Spectrum Disorders (ASD) are lifelong neurodevelopmental disorders that manifest as impairments in behavior, communication and social interaction accompanied by restricted and repetitive interest and possible sensory disturbances usually appearing in early childhood [1]. Parents are often faced with the real challenge resulting from the intense care demands of a child with ASD and its subsequent chronic fatigue [2]. The management of the autistic symptoms and the problematic behaviors of a child with ASD [3] lead to a situation that is difficult to manage. A situation that generally compromises the qualities of marital relations [4] and subsequently leads to serious marital conflicts [5]. Some studies have shown that mothers of a child with ASD have the highest rates of parental stress and anxiety compared to those of children with other intellectual, developmental, or physical disabilities [6-8]. In addition, these mothers suffer more from depression than those of children with intellectual disabilities (ID), without autism, and those of children with typical developmental disabilities [9,10]. These constraints generate specific needs which are unfortunately generally neglected by health professionals in favor of a more traditional decontextualized vision. To fill these gaps, a qualitative approach makes it possible to describe the experience of people in their natural environment with all its characteristics [11]. There are no Tunisian studies assessing the needs of parents of children with ASD. Hence, identifying parental needs is essential, especially for mothers who are most involved in caring for and educating their children. This study aimed to identify the unmet needs perceived by mothers of a preschool child recently diagnosed with ASD in a Tunisian context.

## Methods

**Study design:** a qualitative phenomenological design was chosen for this study in order to explore and better understand the experience as perceived by mothers following the diagnosis of ASD in their children and the resulting needs. The use of this type of study was justified by the novelty and originality of the research and was deemed the right choice to answer the research question aimed at understanding the needs and perceptions of individuals [12].

**Recruitment of participants:** the selection of participants was made in two stages: firstly, the choice was reasoned according to the research objective from the “database of a child psychiatry consultation” to constitute a preliminary list. In fact, we have chosen the mothers of preschool children recently diagnosed with ASD and confirmed by a “child psychiatrist”. Secondly, the enrolled mothers were randomly selected from the preliminary list and contacted by telephone. The researcher explained the study, its purpose, the modality of participation, and the data collection procedure to the participants and obtained their oral consent. Then, she made an appointment according to the availability of the participants. Data saturation was reached in this study after six interviews, after which no new themes or sub-themes emerged. In total, we reached a sample size of eight mothers.

**Conduct of the study:** the interviews were conducted in dialectal Arabic, the mother tongue of the Tunisian population, and mastered by the researcher, who is bilingual in Arabic and French. The interviews lasted between 30 and 40 minutes. The researcher carried out the data collection in two complementary steps. First, the mother completed a socio-demographic questionnaire. Data was collected through in-depth interview based on semi-structured guide. The guide is made up of three parts as below:

***Reaction at the time of diagnosis announcement:*** (thinking back to when you received your child's diagnosis, what did you think (about diagnosis, psychological support)? What did you feel?).

***Need for information:*** (do you feel well and properly informed about your child diagnosis? Who did you seek information from to understand ASD? Are the explanations sufficient and clear? Do you still feel the need for information?).

***Need for support (from society/ family/husband or wife):*** indeed, according to Giorgi (1997) [13], this method implies that participants should not be guided or influenced in their way of thinking. For this reason, we used open questions centered on how they had experienced the diagnosis and the resulting needs, leaving the mothers to describe their experiences and express themselves freely.

**Data analysis:** the interviews were recorded and transcribed verbatim in Arabic by the researcher herself. Then, the data were reread as many times as necessary before being analyzed following the analysis method of Giorgi [13], which consists of five steps: 1) data collection, which consists of repeatedly reading and then transcribing the verbatim recordings of the interviews to begin to identify the ideas that emerge; 2) reading the transcription: this step is used to delimit the content of the verbatim into sections or “meaning units” and to extract the significant segments; 3) the division of data into meaningful units, it requires several readings to isolate sentences sharing the same idea; 4) the organization and enunciation of data, the researcher bases herself on a spirit of synthesis to formulate the main themes and organize the meanings from a logical order. Finally, 5) a triangulation of the analysis data was set up (two experts compared their analyses with the researcher).

**Ethical considerations:** all of the obtained data were considered as confidential and anonymous. The study protocol conformed to ethical guidelines of the 1975 Declaration of Helsinki, approved by the ethic committee of the Faculty of Medicine of Sousse, number: CEFMS 58/2020.

## Results

**Socio-demographic profile of the participants:** the studied population consisted of eight Tunisian mothers with a mean age of 38.25 ± 3.80 years old, the youngest being 36 and the oldest 47 years old. Each mother had a single child with ASD. Of the 8 children enrolled in the study, five are boys and three are girls. The youngest was 4 years old and the oldest 9 years old; their mean age was 5.75 ± 1.75 years. The diagnosis was made, on average at the age of 3.12 ± 0.35 years. All the children were diagnosed with ASD according to the DSMIV-TR criteria by an experienced child psychiatrist. Regarding the socio-demographic profile, the educational level of mothers varied between secondary and university. The socioeconomic level was middle for 3 participants and low for the other five (housewives) as the Table 1 shows.

**Table 1:** study participant´s characteristics

Mother´s characteristics	
**Maternal Age (Years), M (SD) [min, max]**	38.25 ± 3.80 [36-47]
**Marital status N (%)**	
Divorced/separated	0 (0%)
Married	8 (100%)
**Monthly family income level N (%)**	
Low	5 (62.50%)
Middle	3 (37.50%)
	
**Education level N (%)**	
Primary school	0 (0%)
Secondary school	2 (25%)
University	6 (75%)
**Health Insurance N (%)**	
Yes	6 (75%)
No	2 (25%)
**Child´s characteristics**	
**Child Age (Years), M (SD) [min, max]**	5.75 ± 1.75 [4- 9]
**Child gender N (%)**	
Male	5 (62.50%)
Female	3 (37.50%)
**Age of diagnostic (Years), M (SD) [min, max]**	3.12 ± 0.35 [3-4]
**Number of children N (%)**	
Two children	4 (50%)
Three children	3 (37.50%)
Four children	1 (12.50%)
**Rang of child**	
Elder	4 (50%)
Benjamin	2 (25%)
cadet	2 (25%)

**The emerging themes and sub-themes of the study:** the thematic analysis carried out from the interviews made it possible to identify five themes and 17 subthemes (Table 2): 1) the need for emotional support after the announcement of diagnosis (shock and denial, guilt, emotional distress, concern, coping, family support, support from other parents with ASD), 2) the need to be informed (insufficient information, source of information, and area of information), 3) the need to be trained and guided (behavior with the child, communication with the child and parental self-efficacy), 4) the need for social support (social discrimination, the role of associations) and 5) the financial need (limited resources and financial assistance).

**Table 2:** presentation of themes and sub-themes

Themes	Sub-themes
The need for emotional support after the announcement of diagnosis	The shock and denial
	The guilt
	The emotional distress
	The concern
	The coping
	The family support
	The support from other parents with ASD
The need to be informed	The insufficient information
	The source of information
	The area of information
The need to be trained and guided	The behavior with the child
	The communication with the child
	The parental self-efficacy
The need for social support	The social discrimination
	The role of associations
The financial need	Limited resources
	financial assistance

**The need for emotional support:** the first theme identified in the speeches of the mothers is the need for emotional support. It includes the first feelings engendered by the diagnosis and the coping modality adopted by the mothers. Then, after establishing a certain balance, the mothers were able to identify their expectations in terms of emotional support from the family and the surroundings.

***Shock and denial:*** all the mothers illustrated a difficult acceptance. Having a child diagnosed with ASD is still considered a taboo subject to be hidden by society. All mothers expressed denial and refusal of the diagnosis of ASD at the time of family diagnosis:

*"I was shocked… I felt the collapse […] I swear to you I lost all my bearings […] I left almost unconscious of what surrounds me […] I was in shock […] (LI)*.

The refusal concerns, on the one hand, the reality of having a child with autism: on the other hand, *"I never imagined that my child would be autistic"*(MY).

This reality, poorly accepted, gives rise to a feeling of internal conflict and tearing:

*"Because I was always in internal conflict […] always something that refused to accept the reality that my child had ASD"*.(OB).

The refusal goes beyond feelings to influence the behavior of mothers with their children. Indeed, the rejection can affect the child in himself and hinder benevolent attitudes toward him:

*"I couldn't come to terms with the diagnosis. I easily got angry (irritability) I was so shocked, I lost balance in my behavior with him. […] I said I shouldn't have given birth to him."*(HR)

***The guilt feeling:*** an expression of feelings of guilt and responsibility for the child's situation finds himself. The mothers describe this feeling in these terms:

*"I feel guilty, a bitter feeling that I want to get rid of these thoughts and I don't know how"*(LI)

***The distress:*** moms acknowledged their poor psychological well-being after their children's diagnosis. In this sense, our participants expressed significant exhaustion and fatigue, even collapse. This importance is reflected in the frequent use and repetition of the words “tired, exhausted, suffocated, collapse” and the facial expressions of the mothers. And their tears.

*"I have a hard time, a feeling of terrible pain in the depth of my soul. I need someone to help me, to understand me"*(HN)

***The concern/worries:*** generally, worry accentuates feelings of sadness or even depression. It is the fear of tomorrow, the future of the child. A question in front of which the mothers remain frozen full of fear and anguish.

*"I'm afraid of dark thoughts. Another day what his future will be?"* (MY)

***The coping:*** the coping strategies developed by mothers represent one of the main tools that allow them to adapt to the different stressful situations induced by the diagnosis of ASD. Religion strongly affects coping, often coloring any sense of hope and acceptance expressed by mothers, testifying to the sacred relationship that unites them to religion.

*"[…] I know that God is going to help me […] he will send me someone who will help me, guide me, reach out to me […] my daughter would one day be like all other children"*.(HM)

In addition, coping strategies focused on the problem are well used and are expressed from the search for the solution on the part of mothers to meet needs that arose following the announcement of the diagnosis of autism.

*"I scoured the Internet looking for answers to my questions […] I looked up what autism is? How should I behave with my child, and how should I help him?"*(MC).

***Family support:*** family support was identified in the statements of a few participants (n=3), particularly from their mothers and brothers:

*"My brother helped me a lot until now he helps me. My husband cannot take care of my child, on the other hand, my brother takes good care of him, he walks him, he accompanies him to supermarkets."*(HY)

On the other hand, the spouse's support is rarely identified because the shock often alters his psychological state. He needs help:

*"My child's condition has caused me many marital problems. I want him (her husband) to understand me"*.(LI)

**Need for information:** this theme includes mothers' expressions regarding the lack of information, the need for information, and an active search for information, emphasizing its source and domain. This need, which has not been poorly met, generates stress and anxiety among mothers.

***Insufficient information:*** in reaction to the ignorance or lack of knowledge that prevailed when the diagnosis was announced, an active “information seeking” movement occurred for all the mothers encountered (n=8).

*"I did not find answers to my questions. I needed to know more about what is this disease? Why my child? What did I do to cause my child to have this disease? how can I help him?"*(HR).

***Source of information:*** curious mothers address this request, in the first instance, to doctors and competent professionals. But unfortunately, the short time of the interview is insufficient to get all the desired answers.

*"The consultations were not long as I wanted and were not enough. […] I was bombarded with questions. I wanted to know everything about autism which will threaten my daughter's life and her development"*.(HM)

Faced with this situation of non-satisfaction, mothers are looking for an alternative. In our case, the use of the Internet as a source of information was confirmed by all the participants to seek answers to their questions,

*"On the internet, there are oceans of information: what is suitable for my daughter and what is not, and I cannot differentiate between them and choose what is effective and compatible with her condition, but whoever is drowning clings to a straw (Tunisian proverb)."*(LI)

***Information domains:*** the need for information encompasses all dimensions of autism; for them, it is an unusual notion, new and not understood, which arouses the curiosity of mothers to find out what autism is? Its causes? Why my child? How do I communicate with him, and how do I manage his crises?

*"[*…*] I browsed the internet, I tried to discover and find out what this autism is"*.(LI)

*"I want to know, above all, how I should behave with him when he is angry and becomes agitated and violent. How to calm him down? i.e. how I communicate with him"*(MC)

Mothers, in particular with a child of school age, have expressed a need for information concerning the school inclusion of their children.

*"The steps to follow, the papers to prepare, and the schools that accept the inclusion of an autistic child and what will become of our child with normal children, of course, they will not have the same level of mental and intellectual development. And the teachers should have had training to understand our children well"*(HM).

**Need guidance:** this theme includes any expression of the need for support and guidance in the different aspects of behavior management, communication, autonomy, and learning. The mothers expressed a need for advice and support in behavior management, particularly at the time of their child's crisis:

*"I didn't get any guidance or support… I can't deal with his crises… he gets restless, he hits, he breaks… I needed someone to help me understand why he is rebelling and how I can absorb this storm"*.(HY)

**Communications:** *"I want to help him acquire new social skills, even conduct a small dialogue correctly so that he can even buy a product saying bread, for example, I find it difficult to explain to him and make him understand."*(MC)

**Self-efficacy:** the feeling of parental self-efficacy refers to the perception that a person develops in their role as a parent. In this sense, motivation and a sense of responsibility are noted in all mothers. Indeed, the recourse and the search for information and a follow-up with the child psychiatrist, and the search for associations express images of the parents' will to solve the problems and seek solutions. Mothers experience high motivation and a great sense of responsibility towards their children.

*"[…] I promise you that I will execute it. I will do the impossible for my daughter, but only show me how to do this what I ask, I want to help my daughter, I want to save her, and I can save her […]"*. (LI).

**Social support:** this theme focuses on society's view and the discrimination felt by society's mothers and their children with ASD, and the importance of raising awareness in the community about ASD. The mothers said they were isolated:

*"I don't leave the house, neither to visit nor for marriage or dinner. I have no taste. I try not to come into contact with people. I hate it when people start questioning my daughter's condition or making rude remarks […]"*. (HN)

Also, mothers have expressed a need to receive help from associations, but they do not have the correct information or guidance to contact them:

*"I looked for associations, but I did not have the right information and guidance. I tried, but I couldn't find the right person to guide me."*(LI)

**Financial need:** this is the need most mentioned by all the participants (n=8), especially those with low incomes (n=5). In fact, the mothers experience financial difficulties because of the costs associated with their child's ASD, which contrasts with their minimal financial resources.

*"Too many expenses, because of the care costs for my daughter, I ended up bankrupt, I took a bank loan, I sold my inheritance (small piece of land that I inherited from my father) to cover expenses, but what hurts my heart is that I got nothing in return: no improvement. (She was crying) I feel unable to save my daughter."*(HN)

The financial problem has influenced the care of children. In fact, because of the high costs of the centers regarding the financial resources of the family, mothers cannot put their children in private specialized centers:

*"I want to put her in a private specialized center, but I cannot bear the monthly expenses of (150 to 400 dinars per month) apart from the cost of speech therapy sessions and transport. It is impossible with my financial situation"*(LI)

## Discussion

This study revealed an important need for information on the part of mothers. This need arises directly after the announcement of the diagnosis: mothers who lacked information on ASD begin to seek information. This need has been documented by previous studies [14,15]. First, they turn to professionals involved in the diagnosis and management of their children with ASD [16]. Unfortunately, these were often short interviews focused on assessing the child's condition without adequately meeting the information needs of the mothers. Indeed, the mothers expressed their disappointment with the information provided by the professional, considered both insufficient and unsatisfactory. Which has been reported by other studies in different contexts [17,18].

The diagnosis announcement is considered as “instant of crisis” when parents who report feelings of shock, denial, and loss are more exposed to stress and are at risk of developing psychological disorders. However, an adaptive response such as acceptance is correlated with better mental health [19]. That it is important to communicate with a caregiver who listens and conveys a sense of hope when announcing the diagnosis [20]. Therefore, Hennel *et al*. [21] recommend that the child psychiatrist consider the provision of personalized and timely family support, over more than one visit following a written “autism action plan” and specific to the parents' identified needs, may improve understanding, satisfaction, and acceptance of the diagnosis.

As a result, mothers have turned to other sources of information such as internet blogs that are not always verified or scientifically based. This corroborates the study by Georgette *et al*. [22], who reports that parents of children with ASD often use the Internet to search for information. In addition, they refer to discussion forums to confide in, seek advice or help from other parents who are experiencing the same reality [23]. This information concerns, according to the declarations of the mothers, the nature of autism (what is autism?), its causes (why my child?), and the treatment (how to help my child to recover, and manage his behavior?). The same findings were identified in a qualitative study with seven mothers in a Saudi context [24] and others [25].

Indeed, there is a wealth of information available online for parents of children with ASD. Faced with this scourge of information, mothers do not know how to distinguish good information from bad and what suits them and what does not suit them. Moreover, most of the information available in Arabic, on blogs and social networks often lacks scientific basis. Therefore, it is essential to popularize useful information based on scientifically proven research by academic research centers and service providers in the field of autism [26]. This study also highlighted the needs of mothers in terms of training to deal with the behavioral problems of their children. Mothers feel lost and unable to manage the challenging behaviors of their children with ASD. They insist on their need for accompaniment and support in terms of behavior management, communication and autonomy. This is consistent with the findings of Hansen *et al*. [27], in which training for parents of children with ASD was strongly recommended to improve parents' communication and behavior management skills.

Moreover, the shock and denial experienced after the announcement of the diagnosis are clearly expressed by all the mothers, which has been documented by Poirier [28]. Moreover, the literature reports a high level of stress in the parents since the announcement of the diagnosis [29]. Coping begins with seeking formal social support or that of a caregiver. These support networks that are essential to help parents and meet their needs are not always available or adequate [30]. Deprived even of family support in most cases, mothers were overwhelmed by feelings of rejection, guilt and responsibility for the child's illness. This observation was noted in the study by Alqahtani [31]; although the scientific community denied the parenting cause of autism decades ago, the feeling of guilt persists in the parenting community. Finally, if acceptance does occur, it appears to be based on coping centered on religion, which looms large in studying population. This strategy promotes acceptance since having a child with ASD is defined by the word “Ibtilee” which is considered a test of God to measure patience. The “ibtilee” belief generates resignation and acceptance [31].

In this case, the recourse to religion is explained both by the nature of Tunisian culture. This study highlighted a financial need mentioned by all the mothers. The financial overload is explained by the imbalance between, on the one hand, the costs of the expenses of the care of the child with ASD (consultation of the child psychiatrist, speech therapy sessions, center…), and on the other hand by the socio-economic level of the parents (generally medium or low) [27]. Faced with several limitations observed in terms of social support, parents´ resort to individual solutions such as the sale of assets and assistance to the family. The need for intervention by society and associations is accentuated. Moreover, mothers suffer from social stigmatization due to the behavior of children with ASD which is often interpreted by people as having a bad education, making parents feel even more guilty [32], accentuating the loneliness of children and altering, among other things, the quality of friendship with others. These results were found in the study by Lasgaard *et al*. [33]: although all children with autism report having at least one friend, the quality of their friendships is lower in terms of camaraderie, safety and mutual support [33].

The results confirm the need to raise awareness in society and to integrate, support and accompany parents, which will allow them to develop a good sense of well-being and parental competence [34]. Finally, this study highlights the importance of taking into consideration parents' needs in information, coping and guidance when announcing the diagnosis by the child psychiatrist. Hence, the development of a specific action plan to accompany and support parents through caregivers (child psychiatrists, researchers, educators, nurses, speech therapists, etc.) is highly recommended.

**Limitations:** the two main limitations of the study were its relatively small sample size and its short interviews duration of ruffly 30 to 40 min. The qualitative nature of this study allowed us to focus directly on the parents' discourse allowing the access with ease to various encountered-challenges and needs born as a result of a child being diagnosed with ASD. The opted semi-structured interview is the dynamic tool that helps participants freely express themselves and recount as credibly as possible the experience as they perceived it. This study is unique and innovative in its subject and context. Indeed, to our knowledge, no qualitative study has aimed to describe and explore the experiences and challenges of mothers raising a child with autism spectrum disorder in a Tunisian or African context.

## Conclusion

To sum up, this study has found that preschooler child ASD diagnosis triggers a set of needs to their mothers. Indeed, they experienced many challenges and unmet demands, particularly in terms of access to information, training, social and financial support. Study results help to better understand the parenting challenges faced by mothers and highlight the need to develop adequate support strategies for mothers to help them cope with the child's diagnosis and better meet the needs of their families. Thing that it has implications not only for the family but on the whole of the society since the family is only one cell of society´s tissue and the world in general.

### What is known about this topic

- ASD impacts the lives of parents who face challenges that create a battery of needs after a child is diagnosed with ASD.

### What this study adds

- The study documents for the first time the needs and challenges of parents in an African context (Tunisia);
- The study also highlights the experience of parent of children recently diagnosed with ASD;
- Our results may lead as references to help build strong scientific based training program that will take in consideration the real needs of parents.
